# Supplementary material for: A Comparative Assessment of Agronomic and Baking Qualities of Modern/Old Varieties and Landraces of Wheat Grown in Calabria (Italy)
Source: Foods. 2022 Aug 6;11(15):2359. doi: 10.3390/foods11152359 (PMC9368158; doi:10.3390/foods11152359)
Supplement: Supplementary file 1 [file foods-11-02359-s001.zip › foods-1839317-supplementary.pdf]

SUPPLEMENTARY MATERIALS

Table S1. Crumb colour characteristics.

	L*	***a****	***b****
**Genotype**	***	***	***
Altamira	74.6 ± 1.39 [ab]	-1.5 ± 0.20 [cd]	12.8 ± 1.11 [cd]
Bologna	73.4 ± 1.85 [bc]	-1.3 ± 0.25 [bc]	12.3 ± 1.26 [d]
Solehio	75.3 ± 0.74 [ab]	-1.8 ± 0.39 [d]	14.0 ± 1.48 [bc]
PR22R58	73.4 ± 1.69 [bc]	-1.6 ± 0.55 [d]	15.5 ± 2.58 [b]
Abbondanza	75.9 ± 2.25 [a]	-1.1 ± 0.03 [b]	10.0 ± 0.33 [e]
Rosia	71.6 ± 0.92 [cd]	-1.1 ± 0.30 [b]	10.5 ± 1.39 [e]
Mazzancoio	69.4 ± 4.72 [d]	0.4 ± 0.03 [a]	18.5 ± 1.01 [a]
**Location**	***	ns	ns
Rombiolo	72.6 ± [b]	-1.1 ± 0.68 [a]	13.1 ± 2.81 [a]
Maierato	74.1 ± [a]	-1.2 ± 0.77 [a]	13.6 ± 3.35 [a]
**Variety**			
Modern	74.1 ± 1.65	-1.5 ± 0.41	13.6 ± 2.07
Old	75.9 ± 2.25	-1.1 ± 0.03	10.0 ± 0.33
Landrace	70.5 ± 3.51	-0.4 ± 0.78	14.5 ± 4.24

*L**, lightness; *a**, red to green coordinate; *b**, yellow to blue coordinate. Mean values are reported for each variable. Different letters in the same column indicate significant differences according to Tukey HSD test at $p \leq 0.05$. Different letters in the same column indicate significant differences according to Tukey HSD test at $p \leq 0.05$, while ***, ns, indicate the following levels of significance for each ANOVA model: 0.001, not significant Only the mean values ± standard deviation are shown for the variety component for descriptive purposes.

Table S2. Crust colour characteristics.

	*L**	*a**	*b**
**Genotype**	***	***	***
Altamira	42.5 ± 6.57 [ab]	13.7 ± 3.03 [a]	21.6 ± 7.89 [abc]
Bologna	44.8 ± 6.76 [ab]	14.2 ± 1.94 [a]	23.7 ± 6.93 [ab]
Solehio	43.0 ± 4.56 [ab]	14.9 ± 1.11 [a]	22.7 ± 4.78 [ab]
PR22R58	44.3 ± 3.93 [ab]	15.2 ± 1.12 [a]	24.1 ± 4.70 [ab]
Abbondanza	47.8 ± 2.50 [a]	14.9 ± 1.04 [a]	27.2 ± 2.47 [a]
Rosia	39.3 ± 3.20 [bc]	13.4 ± 2.32 [ab]	18.5 ± 5.09 [bc]
Mazzancoio	36.6 ± 2.23 [c]	11.2 ± 0.76 [b]	15.0 ± 1.07 [c]
**Location**	ns	ns	ns
Rombiolo	42.5 ± 5.80 [a]	13.8 ± 1.88 [a]	21.6 ± 6.27 [a]
Maierato	42.8 ± 5.46 [a]	14.0 ± 2.40 [a]	22.0 ± 6.25 [a]
**Variety**			
Modern	43.7 ± 5.49	14.5 ± 1.99	23.0 ± 6.11
Old	47.8 ± 2.50	14.9 ± 1.04	27.2 ± 2.47
Landrace	37.9 ± 3.04	12.3 ± 2.04	16.7 ± 4.00

*L**, lightness; *a**, red to green coordinate; *b**, yellow to blue coordinate. Mean values are reported for each variable. Different letters in the same column indicate significant differences according to Tukey HSD test at $p \le 0.05$. Different letters in the same column indicate significant differences according to Tukey HSD test at $p \le 0.05$, while ***, ns, indicate the following levels of significance for each ANOVA model: 0.001, not significant Only the mean values ± standard deviation are shown for the variety component for descriptive purposes.

Table S3. Full list of R packages used for data analysis, manipulation and graphic representation.

Function	R package	Reference
Base function	"Base functions of R"	[32]
Data wrangling	"dplyr"	[33]
Multiple paired comparisons	"multcompView"	[34]
Correlation computation and representation	"ggstatsplot"	[35]
PCA	"FactoMineR"	[36]
PCA	"factoextra"	[37]
Graphic visualization	"ggplot2"	[38]
Graphic visualization	"ggthemes"	[39]
Graphic visualization	"ggsci"	[40]
Data exportation	"rio"	[41]

Table S4. Details on principal components values and variance.

Principal component	Eigenvalue	% of variance	Cumulative % of variance
1	9.861363838	44.82438108	44.82438108
2	5.311577534	24.14353424	68.96791533
3	3.150107433	14.31867015	83.28658548
4	0.898434333	4.083792424	87.3703779
5	0.752893903	3.422245015	90.79262292
6	0.466842849	2.122012951	92.91463587
7	0.321507338	1.461396992	94.37603286
8	0.262023458	1.19101572	95.56704858
9	0.213776555	0.971711612	96.53876019
10	0.20371589	0.925981319	97.46474151
11	0.128778232	0.585355602	98.05009711
12	0.122624863	0.557385741	98.60748285
13	0.074637497	0.33926135	98.9467442
14	0.071806507	0.326393213	99.27313742
15	0.0641448	0.291567271	99.56470469
16	0.033322182	0.151464463	99.71616915
17	0.027324791	0.124203597	99.84037275
18	0.015204307	0.069110487	99.90948323
19	0.012063103	0.054832286	99.96431552
20	0.003922335	0.017828795	99.98214432
21	0.00231914	0.010541545	99.99268586
22	0.001609111	0.00731414	100

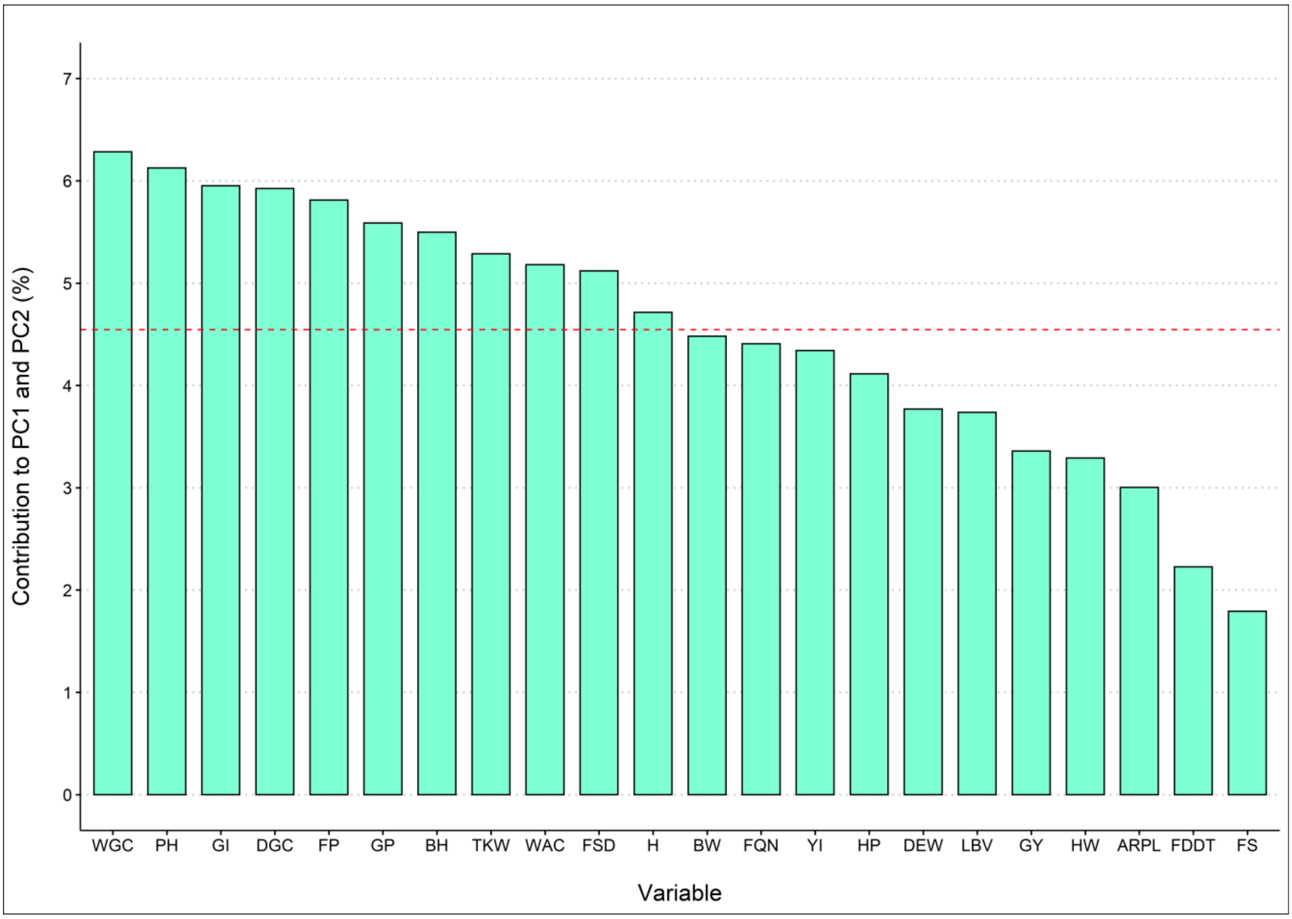

Figure S1. Contribution of variables to PC1 and PC2.

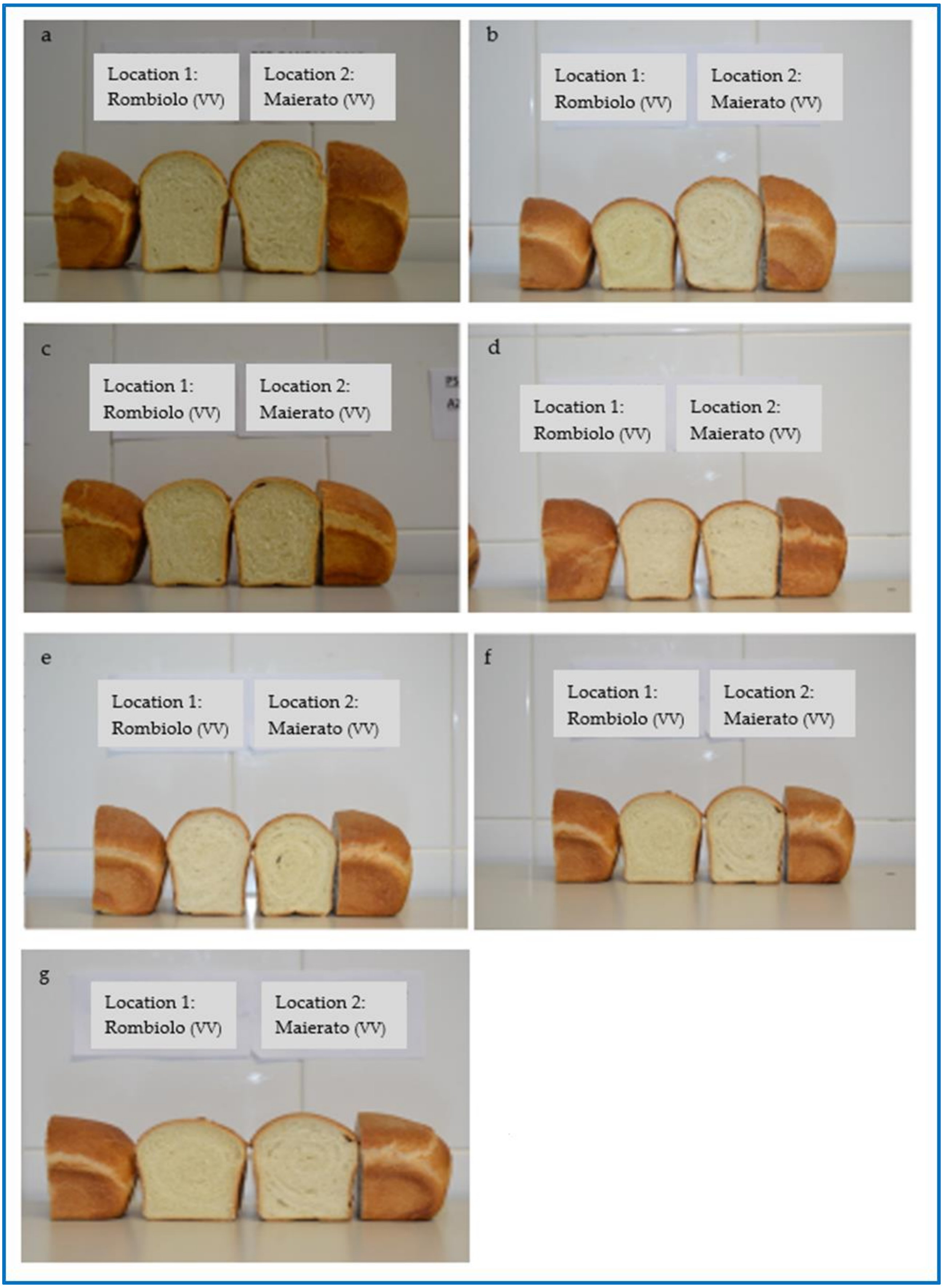

Figure S2. Representation of the different breads obtained using the flours from the varieties under study in the two localities. (**a**) Abbondanza; (**b**) PR22R58; (**c**) Altamira; (**d**) Rosia; (**e**) Bologna; (**f**) Solehio; (**g**) Mazzancoio.
